# Changes in deep neck muscle length from the neutral to forward head posture. A cadaveric study using Thiel cadavers

**DOI:** 10.1002/ca.23834

**Published:** 2022-01-25

**Authors:** Guohao Lin, Weijie Wang, Tracey Wilkinson

**Affiliations:** ^1^ Centre for Anatomy and Human Identification School of Science and Engineering, University of Dundee Dundee UK; ^2^ Department of Orthopaedic and Trauma Surgery School of Medicine, University of Dundee Dundee UK

**Keywords:** cervical spine, deep neck muscles, forward head posture, muscle length change

## Abstract

Forward head posture (FHP) is one of the most common postural deviations. Deep neck muscle imbalance of individuals with FHP is of primary concern in clinical rehabilitation. However, there is scarce quantitative research on changes in deep neck muscle length with the head moving forward. This study aimed to investigate changes in deep neck muscle length with different severity levels of FHP. Six Thiel‐embalmed cadavers (four males and two females) were dissected, and 16 deep neck muscles in each cadaver were modeled by a MicroScribe 3D Digitizer in the neutral head posture, slight FHP, and severe FHP. The craniovertebral angle was used to evaluate the degrees of FHP. Quantitative length change of the deep neck muscles was analyzed using Rhinoceros 3D. In slight FHP significant changes in length occurred in four muscles: two shortened (upper semispinalis capitis, rectus capitis posterior minor) and two lengthened (longus capitis, splenius cervicis). In severe FHP all occipital extensors were significantly shortened (10.6 ± 6.4%), except for obliquus capitis superior, and all cervical extensors were significantly lengthened (4.8 ± 3.4%), while longus capitis (occipital flexor) and the superior oblique part of the longus colli (cervical flexor) were lengthened by 8.8 ± 3.8% and 4.2 ± 3.1%, respectively. No significant length change was observed for the axial rotator. This study presents an alternate anatomical insight into the clinical rehabilitation of FHP. Six muscles appear to be important in restoring optimal head posture, with improvements in FHP being related to interventions associated with the occipital and cervical extensors.

## 1 INTRODUCTION

Modern lifestyles and work requirements mean that many people spend long periods of time looking at screens on electronic devices, often resulting in awkward postures (Toh et al., 2017; Yadegaripour et al., 2021). Forward head posture (FHP) is one of the most common postural deviations observed with different FHP severity levels (Mahmoud et al., 2019); individuals with FHP often have an excessively anterior head position relative to the shoulder (Neumann et al., 2017). According to recent reviews on FHP, this postural deviation is associated with neck pain, vestibular deficits, decreased proprioception, abnormal muscle activity, and altered breathing patterns (Mahmoud et al., 2019; Migliarese & White, 2019; Szczygiel et al., 2020).

Muscular imbalance in the craniocervical region is thought to be directly associated with FHP (Kendall et al., 2005; Sikka et al., 2020). Chronic spasm of the anterior neck muscles or forward translation of the head when watching a computer screen, for example, may facilitate FHP (Neumann et al., 2017). Maintaining an imbalanced position may change the functional resting length of muscles, creating a habitual unnatural posture due to the changed length‐tension relationship and proprioceptive feedback (Kendall et al., 2005; Khan et al., 2020; Moustafa et al., 2020).

The deep neck muscles are of primary concern in FHP treatment because of their relatively high muscle spindle density, which underpins the precision of movement and proprioceptive information (Liu et al., 2003). Rehabilitation of the deep neck muscles has been observed to improve stability of head and neck posture, manifested as increased ability to maintain an upright position of the cervical spine (Blomgren et al., 2018; Falla et al., 2007).

There are, however, inadequate quantitative studies on deep neck muscle length changes. Most of the literature provides qualitative descriptions, with only a single study (Khayatzadeh et al., 2017) reporting quantitative data on neck muscle length changes using a computer‐generated model based on fresh‐frozen cadaveric spines. Cartner et al. (2011) cautioned that the mechanical resistance of fresh‐frozen cadaveric specimens decreases with increasing exposure time.

Further quantitative verification is, therefore, needed to inform clinical decision making in the rehabilitation of FHP. Thiel‐embalmed cadavers have lifelike characteristics, enabling the biomechanical characteristics of muscle, such as mechanical strain rate effects of tendons and ligaments, elasticity, and stiffness of muscles, to be evaluated (Joy et al., 2015; Liao et al., 2015; Vollner et al., 2017). The craniovertebral angle (CVA) is widely used in FHP evaluation, having high validity and excellent inter and intra rater reliability (Migliarese & White, 2019; Salahzadeh et al., 2014). The present study investigated the length changes in deep neck muscles from the neutral posture to different FHP severity levels using Thiel cadavers, with the aim of identifying the changing lengths in deep neck muscles and the muscles most affected by FHP.

## 2 MATERIALS AND METHODS

### 2.1 Specimens and preparation

The study was conducted using six Thiel‐embalmed cadavers (four males, two females) with a mean age at death of 86.2 ± 8.7 (71–97) years from the Centre for Anatomy and Human Identification, University of Dundee. Inclusion criteria for the study were that the cadavers had to have intact deep cervical muscles, cervical and thoracic spine; the exclusion criterion was a history of spinal surgery. All cadaveric research was conducted in compliance with relevant anatomical legislation, with all donors having given their consent in accordance with the Anatomy Act (1984) and the Human Tissue (Scotland) Act (2006). This study was approved by the Ethics Committee of the Centre for Anatomy and Human Identification. All donors also gave permission for images to be taken.

Each cadaver was placed in the lateral decubitus position and supported on both sides of the trunk. Wooden “pillows” were used to keep the head, neck and spine in alignment and to maintain a horizontal gaze (i.e., parallel to the ends of the dissecting table) in order to facilitate measurement of the CVA. Regional dissection, by the same investigator, was then carried out in the cervical region to expose the deep cervical muscles. The deep cervical muscles selected for study were 16 muscles in five different functional groups (Table 1).

**TABLE 1:** The deep cervical muscles selected in five functional groups

Functional group	Muscle name
Occipital extensors	Longissimus capitis
	Upper semispinalis capitis
	Lower semispinalis capitis
	Rectus capitis posterior major
	Rectus capitis posterior minor
	Obliquus capitis superior
Occipital flexors	Rectus capitis lateralis
	Rectus capitis anterior
	Longus capitis
Cervical extensors	Longissimus cervicis
	Semispinalis cervicis
	Splenius cervicis
Cervical flexors	Longus colli (superior oblique)
	Longus colli (vertical)
	Longus colli (inferior oblique)
Axial rotator	Obliquus capitis inferior

**FIGURE 1:**
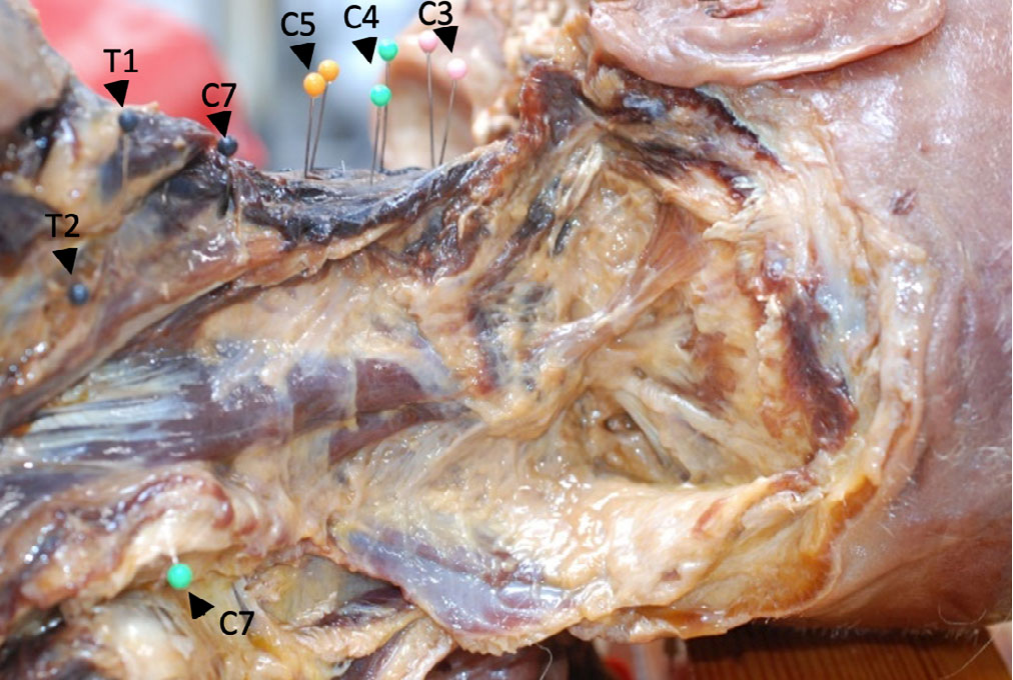
Primary landmarks for locating attachments of neck muscles. The primary bony landmarks were the anterior and posterior tubercles of the transverse processes of C3 (pink), C4 (green), and C5 (yellow), the transverse processes of C7, T1, and T2 (black), the spinous process of C7 (green), and the center point of the anterior surface of the T3 vertebral body (not shown)

Primary landmarks (anterior and posterior tubercles of transverse processes of C3, C4, and C5; transverse processes of C7, T1, and T2; spinous process of C7; center point of the anterior surface of the T3 vertebral body) in the cervical region were marked using pins (Figure 1), enabling the attachments of the deep cervical muscles to be consistently located when measuring muscle lengths.

### 2.2 FHP simulation

**FIGURE 2:**
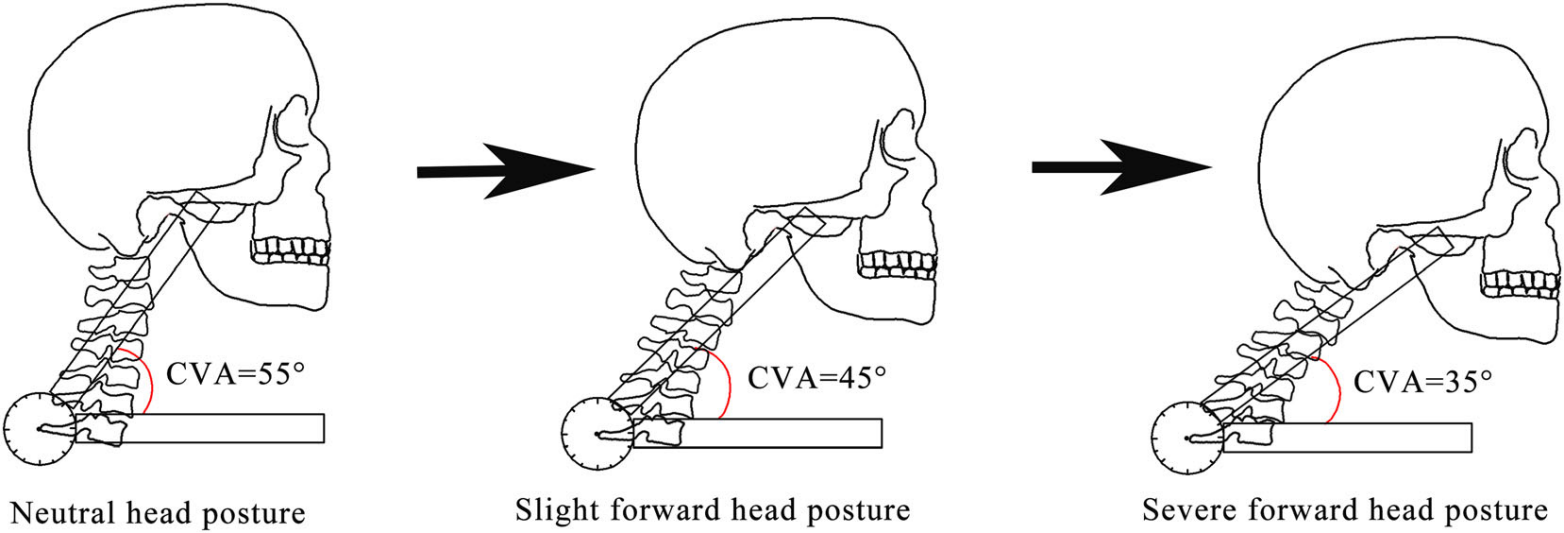
Forward head posture simulation. CVA, craniovertebral angle

As previously noted, CVA is a valid reliable measure to define neutral head posture and FHP (Migliarese & White, 2019; Salahzadeh et al., 2014). It is the angle between a horizontal line passing through the C7 spinous process and a line extending from the tragus of the ear to the C7 spinous process in the sagittal plane (Figure 2). To be consistent with previous studies (Fernandez‐de‐las‐Penas, Alonso‐Blanco, Cuadrado, & Pareja, 2006; Raine & Twomey, 1997; Salahzadeh et al., 2014) the neutral head posture was defined as a CVA of 55°, slight FHP as a CVA of 45° and severe FHP as a CVA of 35°. In each Thiel cadaver, the CVA was measured using a goniometer when the head was moved forward until the predefined CVA was reached. The simulated head postures were fixed using wooden blocks placed anterior and posterior to the head. In all assessments the order of simulation was the same (neutral head posture, slight FHP, severe FHP).

### 2.3 Measurement device and data collection

The lengths of the deep neck muscles were obtained using the MicroScribe 3D Digitizer (Immersion Corporation, California) and Rhinoceros 3D software. The MicroScribe 3D Digitizer was placed in the same position relative to the head for each cadaver. The length of each selected muscle was determined using the stylus of the digitizer by measuring the distance between their proximal and distal attachment points. The muscle attachment sites used in the present study were reported by Khayatzadeh et al. (2017), thereby ensuring that their results could be compared directly with the current study, as well as providing a larger sample size for future research. All selected muscles were considered to pass between their attachments in a straight line, with the length and spatial information being input to the Rhino 3D software. Each measurement was taken twice for each head posture, from which the mean was determined. Any change in muscle length between the neutral head posture and slight or severe FHP was expressed as a percentage of the length in the neutral head posture.

### 2.4 Statistical analysis

Statistical analyses were performed using SPSS v23.0 (IBM SPSS Statistics, Armonk, NY). The normality of the data distributions was assessed using the Shapiro–Wilk test, with test–retest reliability being assessed using the intraclass correlation coefficients (ICC) and 95% confidence intervals (CI): two‐way mixed‐effects model, absolute agreement and single measurement were selected for the ICC analysis. The reliability criteria were set as <0.50 = poor, 0.5 to 0.75 = moderate, 0.75 to 0.9 = good, and >0.90 = excellent (Koo & Li, 2016). One‐way repeated‐measures ANOVA with Fisher's least significant difference (LSD) test as the post‐hoc test was employed to compare the changes in the muscle length between the three head postures. Tests for normality and Mauchly's test of sphericity for the analysis were met. The significance level was set at 0.05.

## 3 RESULTS

### 3.1 Test–retest reliability

Measurements of muscle length in each head posture showed excellent test–retest reliability (ICCs >0.90). In the neutral head posture, the ICC was 0.999 (95% CI = 0.998–0.999, *p* < 0.001); in slight FHP, the ICC was 0.999 (95%CI = 0.998–0.999, *p* < 0.001); in severe FHP, the ICC was 0.998 (95% CI = 0.997–0.999, *p* < 0.001).

### 3.2 Muscle length changes in different head postures

**FIGURE 3:**
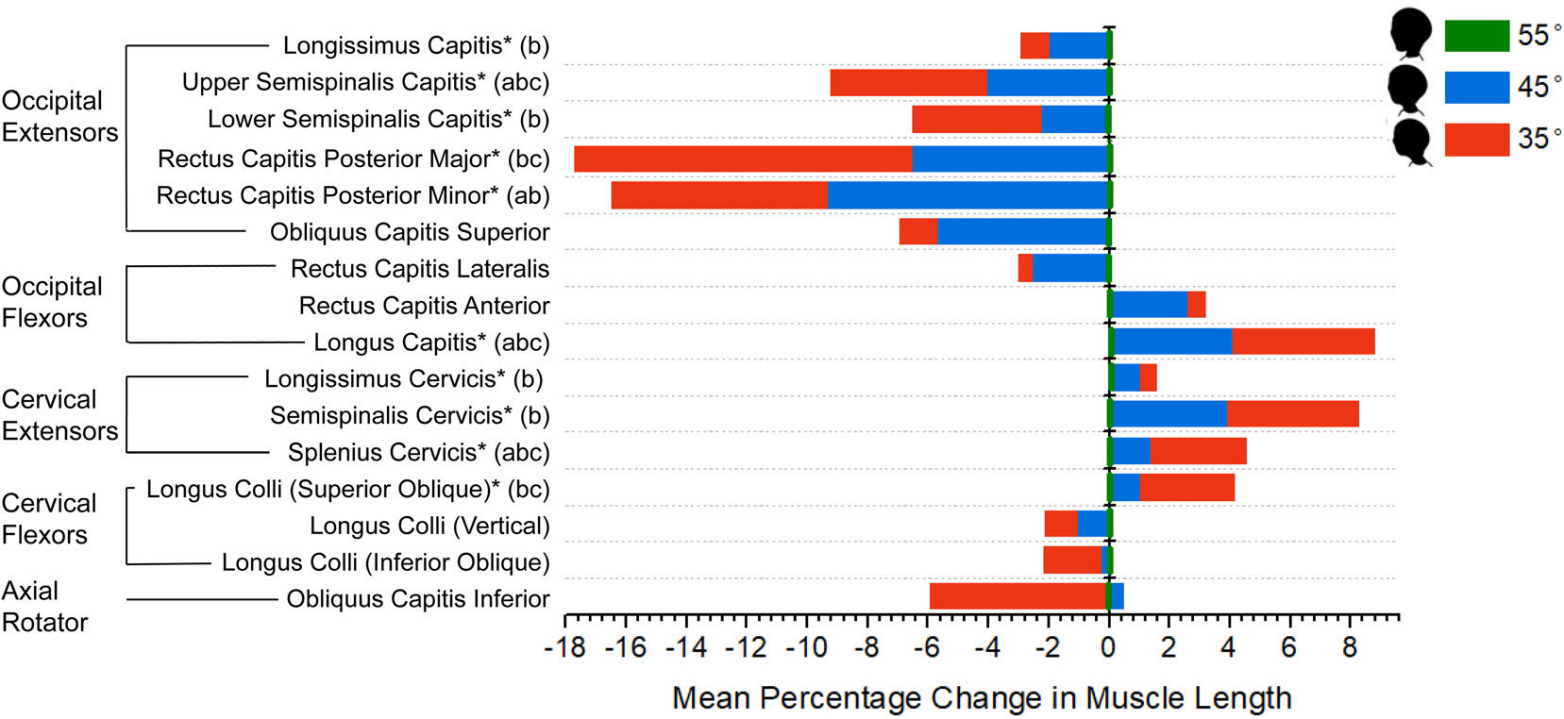
Mean percentage change in deep cervical muscle length from neutral to slight and severe FHP. The changes in blue represent the mean percentage change in muscle length between the neutral head posture and slight FHP; the changes in red are the mean percentage change in muscle length between the neutral head posture and severe FHP. *: *p* < 0.05. a: Significant difference between slight FHP and neutral head posture; b: Significant difference between severe FHP and neutral head posture; c: Significant difference between slight and severe FHP. FHP, forward head posture

The length changes of the deep cervical muscles, the percentage change in length from the neutral posture and results of LSD tests are shown in Table 2 and Figure 3.

**TABLE 2:** Deep neck muscle length and the percentage change in muscle length in each of the three head postures

Functional group	Muscle name	Muscle length (mm) (mean ± SD)					
		55° neutral posture	45° slight FHP	% change (55° vs. 45°)	35° severe FHP	% change (55° vs. 35°)	Effect sizes (*η*_*p*_^*2*^)
Occipital extensors	Longissimus capitis*	81.7 ± 5.4	80.0 ± 4.0	−2.0 ± 2.4	79.3 ± 3.7	−2.9 ± 2.3^b^	0.58
	Upper semispinalis capitis*	100.9 ± 10.8	96.8 ± 10.4	−4.1 ± 2.7^a^ ^,^ ^c^	91.6 ± 10.4	−9.2 ± 4.5^b^ ^,^ ^c^	0.77
	Lower semispinalis capitis*	112.8 ± 14.8	110.0 ± 12.3	−2.3 ± 4.4	105.5 ± 14.6	−6.5 ± 5.8^b^	0.52
	Rectus capitis posterior major*	42.1 ± 6.3	39.2 ± 5.9	−6.5 ± 7.9^c^	34.3 ± 5.0	−17.7 ± 11.0^b^ ^,^ ^c^	0.72
	Rectus capitis posterior minor*	29.1 ± 4.3	26.4 ± 4.1	−9.3 ± 5.3^a^	24.3 ± 3.4	−16.5 ± 4.0^b^	0.82
	Obliquus capitis superior	43.3 ± 5.5	40.9 ± 5.6	−5.7 ± 3.5	40.5 ± 7.4	−7.0 ± 7.1	0.36
Occipital flexors	Rectus capitis lateralis	18.8 ± 3.0	18.3 ± 2.7	−2.6 ± 3.7	18.1 ± 1.9	−3.0 ± 5.8	0.21
	Rectus capitis anterior	30.7 ± 2.7	31.5 ± 2.8	2.6 ± 4.2	31.7 ± 3.4	3.2 ± 6.5	0.14
	Longus capitis*	70.6 ± 3.2	73.5 ± 3.3	4.1 ± 3.1^a^ ^,^ ^c^	76.8 ± 1.7	8.8 ± 3.8^b^ ^,^ ^c^	0.77
Cervical extensors	Longissimus cervicis*	175.5 ± 16.4	177.4 ± 17.8	1.0 ± 1.2	178.3 ± 17.2	1.6 ± 1.5^b^	0.50
	Semispinalis cervicis*	86.9 ± 5.6	90.3 ± 6.4	3.9 ± 4.7	94.1 ± 9.6	8.3 ± 7.5^b^	0.56
	Splenius cervicis*	169.1 ± 9.5	171.5 ± 9.0	1.4 ± 0.7^a^ ^,^ ^c^	176.8 ± 11.6	4.5 ± 2.3^b^ ^,^ ^c^	0.73
Cervical flexors	Longus colli (superior oblique)*	65.9 ± 3.9	66.5 ± 3.6	1.0 ± 2.4^c^	68.6 ± 3.6	4.2 ± 3.1^b^ ^,^ ^c^	0.61
	Longus colli (vertical)	139.9 ± 7.2	138.4 ± 7.9	−1.1 ± 1.2	137.0 ± 7.8	−2.1 ± 2.5	0.45
	Longus colli (inferior oblique)	100.3 ± 10.7	99.9 ± 9.9	−0.3 ± 1.3	98.1 ± 11.0	−2.2 ± 2.0	0.45
Axial rotator	Obliquus capitis inferior	54.6 ± 5.4	54.7 ± 4.4	0.4 ± 5.7	51.1 ± 3.6	−5.9 ± 9.2	0.40

*Note*: *p < 0.05.

^a^ Significant difference between slight FHP and neutral head posture.

^b^ Significant difference between severe FHP and neutral head posture.

^c^ Significant difference between slight and severe FHP.

### 3.3 Occipital extensors

The occipital extensors showed an overall shortening trend as the head moved forward (Table 2 and Figure 3). From the neutral head posture to slight FHP only two muscles significantly shortened, these being upper semispinalis capitis (−4.1 ± 2.7%, *p* = 0.022) and rectus capitis posterior minor (−9.3 ± 5.3%, *p* = 0.01). From the neutral head posture to severe FHP, however, all occipital extensors, except obliquus capitis superior, shortened significantly (*p* < 0.05): the mean shortening was −10.6 ± 6.4%. The most prominent changes in muscle length were observed in rectus capitis posterior major and minor in both slight and severe FHP (Figure 3).

### 3.4 Occipital flexors

Only longus capitis showed a significant change in length (*p* < 0.001), with its length increasing by 4.1 ± 3.2% (*p* = 0.024) in slight FHP, and 8.8 ± 3.8% (*p* = 0.002) in severe FHP (Table 2 and Figure 3). Rectus capitis anterior and rectus capitis lateralis showed no significant length changes, irrespective of increasing FHP severity (Table 2 and Figure 3).

### 3.5 Cervical extensors

The cervical extensors showed an overall lengthening trend as the head moved forward (Table 2 and Figure 3). In slight FHP the splenius cervicis was the only muscle which lengthened significantly (1.4 ± 0.7%, *p* = 0.003), whereas in severe FHP all cervical extensors lengthened by 4.8 ± 3.4% (*p* < 0.05).

### 3.6 Cervical flexors

The three parts of the longus colli exhibited different trends (Table 2 and Figure 3). No significant muscle length changes were observed as the head moved forward to slight FHP. In severe FHP only the superior oblique part of longus colli lengthened significantly (4.2 ± 3.1%, *p* = 0.025).

### 3.7 Axial rotator

No significant length change was observed in obliquus capitis inferior in either slight or severe FHP (Table 2 and Figure 3).

## 4 DISCUSSION

The present study investigated changes in deep neck muscle length between the neutral head posture, defined as a CVA of 55°, and two severity levels of FHP, defined by CVA 45° and 35°, using Thiel cadavers. The observations showed that some muscles were subject to significant length changes when placed in the two simulated FHPs, with consistent patterns of change with increasing FHP severity. These observations provide the basis for developing a new strategy for the rehabilitation of FHP.

### 4.1 Responses of deep neck muscle in different FHP severities

The deep neck muscles respond differently to slight and severe FHP; in slight FHP, four muscles significantly changed their length (Figure 3), with two shortening (upper semispinalis capitis, rectus capitis posterior minor), and two lengthening (longus capitis, splenius cervicis). These four muscles also had the highest effect size values (*η*_*p*_^*2*^ > 0.14). As well as the length changes observed in slight FHP, rectus capitis posterior major and semispinalis cervicis also showed significant length changes in severe FHP. Restoration of the length of these six muscles could be conducive to regaining optimal head posture.

The observations of this study suggest that these six muscles should be the target for rehabilitation of FHP; there is also some supporting evidence from previous studies. Fernandez‐de‐las‐Penas, Alonso‐Blanco, Cuadrado, Gerwin, and Pareja (2006) reported that FHP was associated with the presence of suboccipital myofascial trigger points, which had similar locations to rectus posterior major and minor. Hallgren et al. (2017) were able to verify this, observing that rectus capitis posterior major and minor had significantly increased electromyographic activity in FHP. In an ultrasonographic study (Goodarzi et al., 2018), it was reported that in individuals with FHP the semispinalis capitis exhibited the smallest thickness changes during voluntary isometric contraction. FHP influences the ability to activate semispinalis capitis effectively.

Furthermore, 4‐week deep cervical flexor training was observed to be more effective in improving FHP and neck pain than conventional isometric training (Gupta et al., 2013). Deep cervical flexor training is a low load craniocervical flexion exercise, which mainly strengthens longus capitis and longus colli (Blomgren et al., 2018). A randomized controlled trial investigating the effects of semispinalis cervicis and deep cervical flexor training noted that both improved FHP, neck disability, pain intensity and muscle strength (Suvarnnato et al., 2019). The results of the present study indicate that future FHP rehabilitation research could focus on the six muscles above, that is, upper semispinalis capitis, rectus capitis posterior major/minor, longus capitis, semispinalis cervicis, and splenius cervicis.

### 4.2 Changing patterns of deep neck muscle length and cervical spine movement

FHP is the result of changes in the alignment of the cervical spine, and although this alignment has substantial cervical subtype variability (Daffin et al., 2019), the present study considered FHP to be associated with upper cervical extension and mid‐lower cervical flexion. This is supported by the length pattern changes in the deep neck muscle observed in the current study. From the neutral head posture to the two FHP severity levels, all the shortened muscles were occipital extensors (longissimus capitis, upper/lower semispinalis capitis, rectus posterior capitis major/minor), while the muscles which lengthened were either cervical extensors (longissimus cervicis, semispinalis cervicis, splenius cervicis), occipital flexors (longus capitis) or cervical flexors (superior oblique part of the longus colli). The insertions of the occipital extensors are on the posterior aspect of the skull and upper cervical spine (e.g., the nuchal line, mastoid process and spinous process) (Standring, 2016), suggesting that their shortening controls extension of the head and upper cervical spine. Of the lengthened muscles, longus capitis and the superior oblique part of longus colli will be stretched with extension of the head and upper cervical spine, while the cervical extensors will be stretched with flexion of the mid‐lower cervical region and forward movement of the head in FHP.

The changing patterns of muscle length observed in the current study were similar, but not identical, to the findings of the qualitative studies (Haughie et al., 1995; Simons et al., 1999) and the computerized model study (Khayatzadeh et al., 2017). There is now mounting consensus that during FHP there is shortening of the occipital extensors and lengthening of the occipital flexors. Qualitative descriptions report that the posterior cervical and suboccipital muscles become shortened, and the anterior neck flexors become lengthened (Haughie et al., 1995; Simons et al., 1999), while the computerized‐model study of Khayatzadeh et al. (2017) reported that cervical flexors and occipital extensors shortened, and cervical extensors and occipital flexors lengthened. However, there are conflicting views regarding the cervical extensors and flexors; all cervical extensors were lengthened in the present study, being consistent with the findings of the computerized model, but contrary to qualitative studies. For cervical flexors, the present study showed that only the superior oblique part of longus colli lengthened, with the vertical and inferior oblique parts showing no significant length changes differing from the computerized model study.

It is of importance to note that the findings of the computerized model and qualitative studies differed regarding the cervical flexors; they were shortened in the computerized model and lengthened in the qualitative studies. This difference between cadaveric and computerized model studies may be due to the different samples used and methods employed. For example, Khayatzadeh et al. (2017) simulated FHP by adjusting the displacement of the occiput‐T1 sagittal vertical alignment without controlling the height of specimen; this could have introduced bias, since differing severity levels of FHP at the same displacement of the cervical spine could have been compared. Nevertheless, further investigation is required to address these conflicting findings.

### 4.3 Muscle weakness and the length‐force relationship

Both shortening and lengthening of a muscle would impair its ability to generate muscle force (Goodarzi et al., 2018; Levangie & Norkin, 2011). Based on the length‐force relationship of skeletal muscles, the maximal force generation can only be achieved at the optimal muscle length (Levangie & Norkin, 2011). Consequently, muscles with altered resting lengths may have lower mechanical efficiency and impaired contractile function, reducing their mechanical economy during daily activities (Szczygiel et al., 2020). Goodarzi et al. (2018) reported that maximal voluntary isometric contraction of the neck extensors significantly decreased with increasing FHP severity. Oliveira and Silva (2016) also reported a significant association between neck muscles endurance (deep neck flexor and neck extensor), FHP and neck pain, which support the impact of FHP on muscle contractile function and the length‐force relationship.

### 4.4 New insight into the clinical rehabilitation of FHP

Deep cervical flexor training is a common corrective treatment for FHP by strengthening longus capitis and longus colli (Falla et al., 2007; Gupta et al., 2013; Kim & Kwag, 2016; Lee et al., 2013); however, its clinical efficacy is still controversial. Sikka et al. (2020) reported that a 4‐week deep cervical flexor training program did not correct FHP in adolescents. Subbarayalu and Ameer (2017) found no significant relationship between deep cervical flexor performance and FHP, indicating that deep cervical flexor training would not provide a satisfying therapeutic effect for individuals with FHP. Nevertheless, the theoretical basis of such training is based on research demonstrating an imbalance between the superficial and deep cervical flexor muscles in individuals with neck pain (Falla et al., 2004), rather than individuals with FHP. Moreover, the relationship between FHP and neck pain remains unclear since confounding factors (e.g., age) may play an important role (Mahmoud et al., 2019). Deep cervical flexor training may, therefore, be more effective in alleviating pain than improving FHP (Sikka et al., 2020).

The current findings may provide future directions for improving the clinical rehabilitation of FHP. According to the present study, of the cervical flexors only the superior oblique part of the longus colli was significantly changed (Figure 3). Deep cervical flexor training for longus capitis and longus colli would be less effective for the rehabilitation of FHP. Furthermore, all cervical and most occipital extensors showed significant changes in severe FHP. It is suggested that the therapeutic effects of rehabilitation would be enhanced if additional interventions for these muscle groups were combined with deep cervical flexor training. A combined treatment of suboccipital release and deep cervical flexor training has been shown to improve FHP significantly in short‐term treatment (Kim et al., 2016). Furthermore, semispinalis cervicis training and deep cervical flexor training have also been shown to have a similarly significant effect on improving FHP, neck pain and neck disability compared with usual care (i.e., manual therapy, modality, electrotherapy, stretching and upper‐limb‐strengthening exercises) (Suvarnnato et al., 2019).

Structural correction of cervical sagittal alignment should also be considered in FHP rehabilitation as FHP is commonly associated with spinal malalignment (Shahar & Sayers, 2019). The study by Shahar and Sayers (2019) indicated that the cervical sagittal configuration became more lordotic in patients with FHP after a 12‐week spinal traction intervention, accompanied by reduction of symptoms (e.g., cranio‐cervical stiffness). A randomized controlled trial by Moustafa et al. (2018) also reported positive changes in the cervical sagittal configuration after 10‐week spinal traction among participants with CVA less than 50 degrees. Thus, in order to achieve superior overall outcomes, interventions for deep cervical muscles and cervical sagittal alignment should be considered for the FHP rehabilitation regimen.

### 4.5 Limitations

There are some limitations in the current study. First, the study is a cadaveric study where CVA measures were taken in a side lying position and not in an upright position. Second, the mean age of the cadavers (86.2 ± 8.7 [71–97] years) indicated that degenerative changes would occur in the aging spine (Tetreault et al., 2015). For example, osteoarthritic changes were likely to be present in C5/6 and C6/7 segments (White et al., 2007), which could alter the adjacent cervical mechanics when simulating FHP, thus changing the apparent length of the longer neck muscles. Third, the sample size was relatively small; a few muscles with large effect sizes showed no change in length (Table 2). Future studies should verify these findings with larger sample sizes. Nevertheless, direct cadaveric measurement and the excellent test–retest reliability gives confidence in the observations reported.

This appears to be the first study to investigate changes in deep neck muscle length in FHP using Thiel cadavers. The observations indicate that the changes in deep neck muscle length have regular patterns with increasing FHP severity, with six muscles (upper semispinalis capitis, rectus capitis posterior major/minor, longus capitis, semispinalis cervicis, splenius cervicis) showing substantial and significant length changes in FHP; consequently, the aim of the study was achieved. These findings could provide a new therapeutic strategy for the clinical rehabilitation of FHP by combining additional interventions for the occipital and cervical extensors with existing training programs.
